# The association between klotho and kidney and cardiovascular outcomes: a comprehensive systematic review and meta-analysis

**DOI:** 10.1093/ckj/sfae255

**Published:** 2024-08-23

**Authors:** Mehmet Kanbay, Crischentian Brinza, Lasin Ozbek, Mustafa Guldan, Uluman Sisman, Sidar Copur, Andreea Covic, Dragos-Viorel Scripcariu, Alexandru Burlacu, Adrian Covic

**Affiliations:** Department of Medicine, Division of Nephrology, Koç University School of Medicine, Istanbul, Turkey; Faculty of Medicine, University of Medicine and Pharmacy “Grigore T Popa”, Iasi, Romania; Institute of Cardiovascular Diseases “Prof. Dr George I.M. Georgescu”, Iasi, Romania; Department of Medicine, Koç University School of Medicine, Istanbul, Turkey; Department of Medicine, Koç University School of Medicine, Istanbul, Turkey; Department of Medicine, Koç University School of Medicine, Istanbul, Turkey; Department of Internal Medicine, Koç University School of Medicine, Istanbul, Turkey; Faculty of Medicine, University of Medicine and Pharmacy “Grigore T Popa”, Iasi, Romania; Faculty of Medicine, University of Medicine and Pharmacy “Grigore T Popa”, Iasi, Romania; Faculty of Medicine, University of Medicine and Pharmacy “Grigore T Popa”, Iasi, Romania; Institute of Cardiovascular Diseases “Prof. Dr George I.M. Georgescu”, Iasi, Romania; Faculty of Medicine, University of Medicine and Pharmacy “Grigore T Popa”, Iasi, Romania; Nephrology Clinic, Dialysis, and Renal Transplant Center “C.I. Parhon” University Hospital, Iasi, Romania

**Keywords:** all-cause mortality, cardiovascular mortality, chronic kidney disease, end-stage kidney disease, klotho

## Abstract

**Background:**

Chronic kidney disease (CKD) and end-stage renal disease (ESKD) are significant global health challenges associated with progressive kidney dysfunction and numerous complications, including cardiovascular disease and mortality. This study aims to explore the potential association between plasma klotho levels and various prognostic outcomes in CKD and ESKD, including all-cause mortality, cardiovascular events, metabolic syndrome development and adverse renal events necessitating renal replacement therapies.

**Methods:**

A literature search was conducted through 3 June 2024 using the electronic databases Cochrane Library, Ovid MEDLINE, CINAHL, Web of Science, SCOPUS and PubMed. This systematic review adheres to the Preferred Reporting Items for Systematic Reviews and Meta-Analyses guidelines.

**Results:**

Fourteen studies were included. For all-cause mortality, comparing CKD patients with low versus high klotho levels showed a significant association {odds ratio [OR] 1.81 [95% confidence interval (CI) 1.34–2.44], *P* = .0001}, with substantial heterogeneity (*I*^2^ = 69%). Excluding one study reduced heterogeneity (*I*^2^ = 43%) while maintaining significance [OR 1.97 (95% CI 1.45–2.66), *P* < .0001]. Cardiovascular mortality was higher in patients with low klotho levels [OR 2.11 (95% CI 1.61–2.76), *P* < .00001], with low heterogeneity (*I*^2^ = 25%). Excluding one study eliminated heterogeneity (*I*^2^ = 0%) while maintaining significance [OR 2.39 (95% CI 1.83–3.12), *P* < .00001]. Composite cardiovascular events did not differ significantly between low and high klotho groups [OR 1.51 (95% CI 0.82–2.77), *P* = .18], but with high heterogeneity (*I*^2^ = 72%). Patients with low klotho levels had a higher risk of adverse renal events [OR 2.36 (95% CI 1.37–4.08), *P* = .002], with moderate heterogeneity (*I*^2^ = 61%). Sensitivity analysis reduced heterogeneity (*I*^2^ = 0%) while maintaining significance [OR 3.08 (95% CI 1.96–4.85), *P* < .00001]. Specifically, for ESKD or kidney replacement therapy risk, low klotho levels were associated with an increased risk [OR 2.30 (95% CI 1.26–4.21), *P* = .007]. Similarly, CKD progression risk was higher in patients with lower klotho levels [OR 2.48 (95% CI 1.45–4.23), *P* = .0009].

**Conclusion:**

Lower serum klotho levels serve as a significant predictor of adverse outcomes, including increased risks of all-cause mortality, cardiovascular mortality and progression to end-stage kidney disease among CKD patients.

## INTRODUCTION

Chronic kidney disease (CKD) and end-stage kidney disease (ESKD) represent major public health challenges, affecting millions of individuals globally and leading to significant morbidity and mortality. CKD is characterized by a gradual decline in kidney function, often due to underlying conditions such as diabetes, hypertension and glomerulonephritis. As kidney function deteriorates, the body's ability to maintain fluid and electrolyte balance, excrete waste products and regulate blood pressure becomes increasingly compromised. This decline can progress to ESKD, where kidney function is severely impaired, necessitating renal replacement therapies like dialysis or kidney transplantation. The progression of CKD is often accompanied by various complications, including cardiovascular events, metabolic disorders and a heightened risk of all-cause mortality [1]. Understanding the factors that influence the progression and prognosis of CKD is crucial for improving patient outcomes and developing targeted therapeutic strategies.

The klotho protein was initially discovered by Japanese scientists in spontaneously hypertensive rats in 1997 [2]. In subsequent studies, this protein, a product of the *KL* gene, has emerged as a critical regulator of various physiological processes, including aging, phosphate metabolism and vascular health. Initially identified as an anti-aging factor, klotho exists in both membrane-bound and soluble forms, with the latter circulating in the bloodstream and exerting systemic effects [3]. Transmembrane klotho protein (mklotho) is expressed mainly in choroid plexus epithelial cells of the brain and the distal convoluted tubules of the kidney. The extracellular region of transmembrane Klotho protein can be cleaved by α- and β-secretases and eventually finds its way into blood, urine and cerebrospinal fluid as soluble klotho (sklotho) [4, 5]. In the context of kidney disease, klotho deficiency has been linked to accelerated progression of CKD, cardiovascular complications and higher mortality rates [6–8]. Recent studies have suggested that plasma klotho levels could serve as a biomarker for disease prognosis, providing valuable insights into the underlying mechanisms driving adverse outcomes in CKD and ESKD patients [9–12].

Despite the published literature highlighting the importance of klotho in kidney disease, significant gaps remain in our understanding of how klotho levels specifically influence prognostic outcomes in CKD patients. While several articles have studied the association between klotho deficiency and adverse renal and cardiovascular outcomes, these findings are limited by small sample sizes and varying methodologies, such as different classification methods of the measured klotho levels among different studies. Additionally, the potential of klotho as a reliable biomarker for predicting clinical outcomes has not yet been fully established and its integration into clinical practice remains as a challenge due to the variability in measured klotho levels and the lack of standardized measurement protocols.

Therefore, this meta-analysis aimed to address such gaps by systematically analysing and synthesizing existing data on the association between plasma klotho levels and a range of prognostic outcomes, including all-cause mortality, cardiovascular mortality, cardiovascular events, developing metabolic syndrome, adverse renal events including CKD progression, development of ESKD and initiation of renal replacement therapies. In addition, this meta-analysis seeks to clarify the role of klotho as a prognostic biomarker and determine its potential impact on clinical decision-making, which could lead to more informed therapeutic strategies and improved outcomes for patients with CKD and ESKD.

## MATERIALS AND METHODS

This meta-analysis was conducted in accordance with the Preferred Reporting Items for Systematic Reviews and Meta-Analyses 2020 guidelines [13].

### Data sources and literature search

A comprehensive literature search was conducted through 3 June 2024, using the electronic databases Cochrane Library, Ovid MEDLINE, CINAHL, Web of Science, SCOPUS and PubMed. The search targeted articles related to klotho using various keyword combinations, including ‘klotho,’ ‘chronic kidney disease,’ ‘chronic renal insufficiency,’ ‘end-stage kidney disease,’ ‘renal replacement therapy,’ ‘haemodialysis,’ ‘GFR decline,’ ‘diabetic kidney,’ ‘all-cause mortality,’ ‘cardiovascular mortality,’ ‘cardiovascular event,’ ‘mortality,’ ‘survival’ and ‘metabolic syndrome’. The detailed search strategy and all of the search terms used for each database are presented in Supplementary Table S1. Additionally, a manual search of references in relevant retrieved articles and review articles was conducted to identify studies not captured in the initial search. The resulting literature search was exported to Covidence (Covidence, Melbourne, VIC, Australia. Each article was independently screened by at least two authors (L.O., M.G. and U.S.).

### Study selection process

During the literature search, articles written in all languages were included. Covidence systematic review software was used to filter out duplicate references from different databases at the outset. The eligibility criteria for inclusion in the meta-analysis were as follows: studies involving adult patients (≥18 years of age) with CKD or ESKD, including those receiving haemodialysis or peritoneal dialysis; studies reporting measurements of plasma klotho levels; and studies evaluating prognostic outcomes related to renal, cardiovascular and metabolic issues. Studies were excluded if they involved patients without CKD or ESKD, focused on paediatric patients (age <18 years), were animal studies only, were literature reviews or other non-original research articles or did not include plasma/serum klotho level measurements. Reference filtration and data extraction were conducted independently by the authors. Any conflicts regarding study inclusion in the meta-analysis were resolved by a third author. The quality of studies was assessed using the Newcastle–Ottawa Scale, which awards a maximum total score of 8 points for each study. This scale evaluates three broad domains: selection of study groups (including representativeness of the exposed cohort, selection of the non-exposed cohort, ascertainment of exposure and demonstration that the outcome of interest was not present at the start of the study), comparability of the groups (based on the design or analysis) and ascertainment of outcomes (including assessment of the outcome, follow-up duration and adequacy of cohort follow-up). The summary of the Newcastle–Ottawa Scale assessment of eligible articles can be found in Supplementary Table S2.

### Outcome measures

We assessed the association between klotho levels and various prognostic outcomes, including all-cause mortality, cardiovascular mortality, cardiovascular events, development of metabolic syndrome, adverse renal events such as CKD progression, development of ESKD, glomerular filtration rate (GFR) decline and initiation of renal replacement therapies. Patients and events were categorized into low klotho and high klotho levels based on the cut-offs specified in individual studies, typically the median or the value best predicting adverse events. For studies reporting klotho data in quartiles, the first quartile defined the low klotho group, while the remaining quartiles were combined to form the high klotho group. The primary outcome was the association between plasma klotho levels and various prognostic outcomes mentioned above.

### Statistical analysis

For this meta-analysis, a random effects model was employed to calculate treatment effects, expressed as risk ratios (RRs) with 95% confidence intervals (CIs) for the outcomes. Additionally, hazard ratios (HRs) extracted from study protocols were pooled using a random effects model. A treatment effect was considered significant if *P* < .05.

### Evaluation of heterogeneity and publication bias

Heterogeneity in treatment estimates was assessed using the Cochran Q test and the *I*^2^ statistic, where substantial heterogeneity was defined as values >50% (Cochrane Handbook for Systematic Reviews of Interventions, version 5.3). Data analyses were conducted using Review Manager software (version 5.3, Cochrane Collaboration, London, UK; 2012).

## RESULTS

A systematic search was performed across designated databases, initially yielding 2833 records. Following the exclusion of duplicate publications, a dataset of 1175 references was obtained. These articles underwent evaluation based on predetermined inclusion and exclusion criteria (Supplementary  Fig. S1). Following this screening process, 14 studies met the eligibility criteria and were included in the present analysis (Table 1).

**Table 1:** Baseline characteristics of studies included in the meta-analysis.

Study	Study design	Number of participants/population characteristics	Outcome measures	Key findings	Quality assessment
Cai *et al.* (2021) [37]	Retrospective cohort	Maintenance haemodialysis patients (*n* = 128): • Low sklotho group, ≤567.8 (*n* = 64) • High sklotho group, >567.8 (*n* = 64)	• All-cause mortality • Cardiovascular mortality	• Lower sklotho was linked with higher CVD mortality	High (score: 8)
Chen *et al.* (2024) [14]	Retrospective cohort	Patients who tested for both klotho and vitamin D from a national survey • klotho >848.4 pg/ml (*n* = 4144) • klotho <848.4 pg/ml (*n* = 5762)	• All-cause mortality • Cardiovascular mortality	• Lower klotho is associated with a significant all-cause and CVD mortality risk at serum 25(OH)D <50 nmol/l • Vitamin D metabolism disruption accessed by the combination of low serum 25(OH)D and low klotho was associated with significant all-cause mortality and CVD mortality	High (score: 8)
Edmonston *et al.* (2024) [15]	Prospective cohort	1088 participants from the Chronic Renal Insufficiency Cohort Study	• All-cause mortality • Heart failure hospitalization • Atherosclerotic cardiovascular events • Composite kidney endpoint (sustained 50% decline in eGFR, dialysis, kidney transplantation, or eGFR <15 ml/min/1.73 m^2^)	• Circulating klotho levels were not significantly associated with clinical outcomes in CKD • High klotho levels did not show a significant difference in outcomes compared to low klotho levels	High (score: 8)
Liu *et al.* (2018) [38]	Prospective cohort^a^	112 patients with CKD stages 3–5 30 healthy volunteers as the control group	• Composite endpoint events: serum creatinine doubling, initiation of RRT or death	• Patients with higher sklotho levels had a reduced risk of adverse kidney outcomes during the follow-up period • Cox regression analysis identified low sklotho as an independent risk factor for CKD progression	High (score: 7)
Liu *et al.* (2022) [39]	Prospective cohort	134 maintenance haemodialysis patients: • Low soluble α-klotho group: 68 patients (≤1.15 ng/ml) • High soluble α-klotho group: 66 patients (>1.15 ng/ml)	• 3-year all-cause mortality rate • Overall survival • Cardio-cerebrovascular mortality	• The 3-year all-cause mortality rate was significantly higher in the low soluble α-klotho group compared with the high soluble α-klotho group (33.82% versus 16.67%; *P* = .039). • Cardio-cerebrovascular mortality rates showed a trend towards significance but were not statistically significant between the two groups (26.47% versus 15.15%; *P* = .107)	High (score: 8)
Liu *et al.* (2024) [40]	Retrospective cohort	2418 individuals ages 40–79 years with stage 1–4 CKD	• All-cause mortality, cardiovascular mortality	• Lower klotho levels associated with increased risk of all-cause mortality and cardiovascular mortality compared with higher levels	High (score: 9)
Martins *et al.* (2023) [41]	Prospective cohort	200 haemodialysis patients	• Presence of peripheral vascular disease • Occurrence of cardiovascular events (fatal/non-fatal stroke or myocardial infarction) • All-cause mortality	• Higher levels of α-klotho were associated with a reduced risk of all-cause mortality	High (score: 7)
Memmos *et al.* (2019) [21]	Prospective cohort	Patients on maintenance haemodialysis (*n* = 79) • Low klotho ≤745, *n* = 40 • High klotho >745, *n* = 39	• All-cause mortality • Cardiovascular mortality • Cardiovascular events	• Cumulative survival was insignificantly lower, but cumulative cardiovascular survival and cumulative freedom from the cardiovascular composite outcome were significantly lower in the low-klotho group	High (score: 8)
Otani-Takei *et al.* (2015) [42]	Prospective cohort	Chronic haemodialysis patients (*n* = 63) • Low klotho, <309 pg/ml (*n* = 14) • Middle klotho, ≥309–<449 pg/ml (*n* = 35) • High klotho, ≥449 pg/ml (*n* = 14)	• All-cause mortality • Cardiovascular mortality • Cardiovascular events	• Patients with serum soluble klotho levels below the lower quartile (<309 pg/ml) had significantly higher cardiovascular and all-cause mortality rates	High (score: 8)
Yu *et al.* (2020) [43]	Prospective cohort	Patients with ESKD on maintenance haemodialysis (*n* = 211) • Low klotho <1.34 ng/ml (*n* = 105) • High klotho ≥1.34 ng/ml (*n* = 106)	• All-cause mortality • Cardiovascular mortality • Cardiovascular events	• Lower sklotho levels had a greater risk of CV events and all-cause mortality • Low sklotho level was strongly correlated with CV morbidity and all-cause mortality	High (score: 8)
Zheng *et al.* (2018) [22]	Prospective cohort	Patients with ESKD on maintenance haemodialysis (*n* = 128) • Low sklotho group (sklotho level <401.96 ng/l) (*n* = 70) • High sklotho group (sklotho level >401.96 ng/l) (*n* = 58)	• All-cause mortality • Cardiovascular mortality • Cardiovascular events • CKD progression	• The coronary artery calcification (CAC) score of patients in the high sklotho group was significantly lower than that of the low sklotho group • sklotho level (*P* < .001) was an independent risk factor for CAC progression • Survival time of the patients in the low sklotho group was significantly lower than that of the high sklotho group	High (score: 7)
Kim *et al.* (2018) [44]	Retrospective cohort	Patients with CKD stage 1–5 without class III and IV heart failure (*n* = 2101) • klotho levels: 1st quartile (*n* = 524) 2nd quartile (*n* = 528) 3rd quartile (*n* = 523) 4th quartile (*n* = 526)	• Cardiovascular events • GFR decline	• Advanced CKD stages were associated with lower serum klotho levels • Ascending quartiles of klotho were significantly associated with decreased left ventricular mass index • Serum klotho had a significant inverse association with left ventricular mass index • No significant association between sklotho and brachial-to-ankle pulse wave velocity	Moderate (score: 6)
Fountoulakis *et al.* (2018) [15]	Prospective cohort	Patients with type 2 diabetes and evidence of diabetic kidney disease (*n* = 101) • sklotho >204.4 pg/ml (*n* = 51) • sklotho <204.4 pg/ml (*n* = 50)	• CKD progression • GFR decline	• Patients with residual microalbuminuria despite renin–angiotensin system blockade had significantly lower levels of sklotho compared with patients without microalbuminuria • Higher sklotho levels reduced the incidence of renal function decline • sklotho below the median of 204.4 pg/ml had nearly a 4-fold higher cumulative incidence of renal function decline compared with those above the median	High (score: 8)
Buiten *et al.* (2014) [45]	Prospective cohort	Dialysis patients and left ventricular dysfunction (*n* = 127) • klotho <460 pg/ml (*n* = 63) • klotho >460 pg/ml (*n* = 64)	• Cardiovascular events	• Low sklotho (<460 pg/ml) showed significantly more coronary artery disease and left ventricular dysfunction • sklotho was not independently associated with the presence of CVD or abdominal aorta calcification	Moderate (score: 6)

a This study has an additional cross-sectional component.

CVD: cardiovascular disease.

### Klotho and all-cause mortality

**Figure 1:**
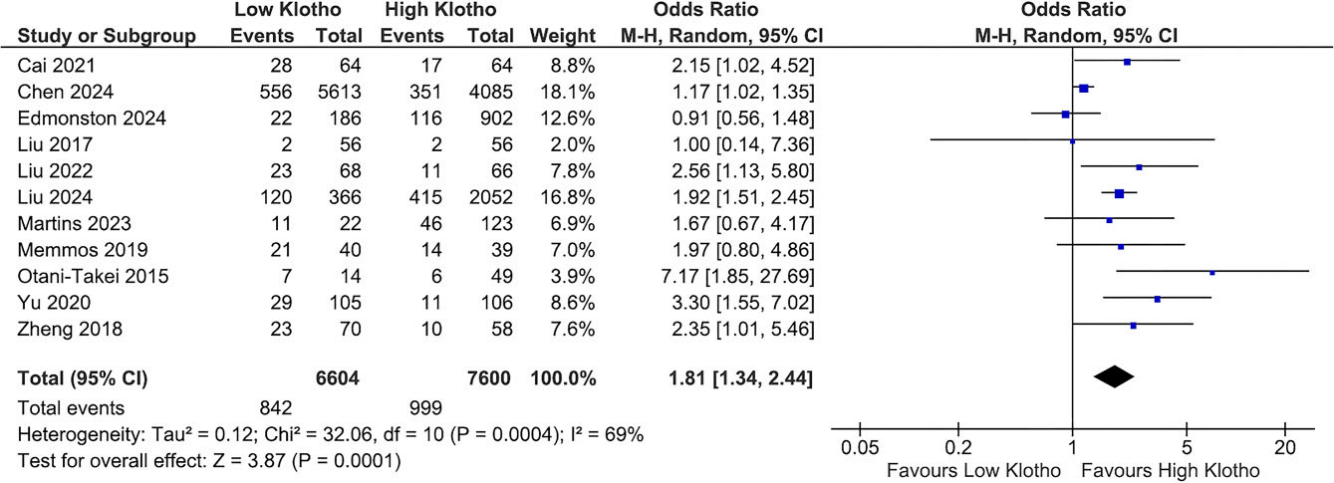
Klotho values and all-cause mortality.

Concerning all-cause mortality, 11 studies compared CKD patients with low and high klotho levels (Fig. 1). The pooled analysis demonstrated that lower klotho levels were significantly associated with increased all-cause mortality compared with higher levels [OR 1.81 (95% CI 1.34–2.44), *P* = .0001], although there was considerable heterogeneity among the studies (*I*^2^ = 69%). Sensitivity analysis, excluding the study by Chen *et al.* [14], reduced heterogeneity (*I*^2^ = 43%) while maintaining a statistically significant overall effect [OR 1.97 (95% CI 1.45–2.66), *P* < .0001].

### Klotho and cardiovascular mortality

**Figure 2:**
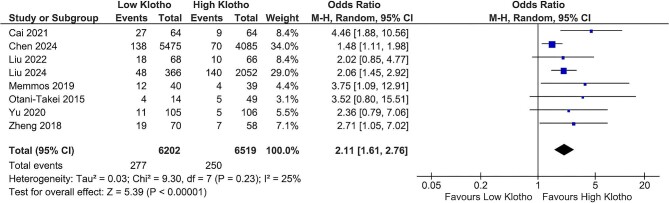
Klotho values and cardiovascular mortality.

Cardiovascular mortality occurred more frequently in patients with low klotho values compared with those with high klotho values (4.4% versus 3.8%, respectively). The risk of cardiovascular mortality was more than doubled in individuals with low klotho values [OR 2.11 (95% CI 1.61–2.76), *P* < .00001], with relatively low heterogeneity observed (*I*^2^ = 25%) (Fig. 2). Exclusion of the study by Chen *et al.* [14] resulted in a further reduction of heterogeneity (*I*^2^ = 0%) while maintaining a significant overall effect [OR 2.39 (95% CI 1.83–3.12), *P* < .00001].

### Klotho and cardiovascular events

**Figure 3:**
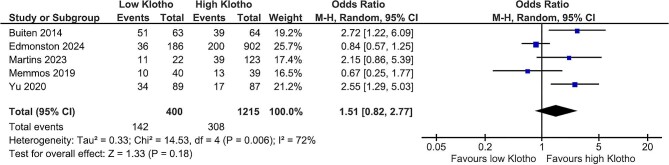
Klotho values and composite cardiovascular events.

As a secondary outcome, composite cardiovascular events including heart failure hospitalizations, coronary artery disease, myocardial infarction, stroke and peripheral artery disease showed comparable rates between both klotho groups [OR 1.51 (95% CI 0.82–2.77), *P* = .18] (Fig. 3). However, the effect was limited by the high heterogeneity (*I*^2^ = 72%).

### Klotho and renal events

**Figure 4:**
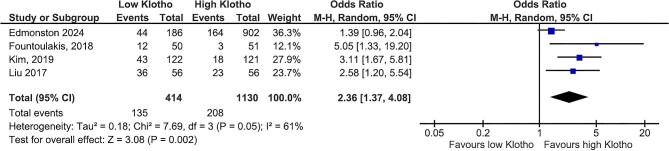
Klotho values and the composite of ESKD, KRT or CKD progression.

Low klotho levels were linked to a significantly increased risk of adverse renal events. The composite endpoint of ESKD, kidney replacement therapy (KRT), or CKD progression was more than 2-fold higher in patients with low klotho values [OR 2.36 (95% CI 1.37–4.08), *P* = .002] (Fig. 4). However, this analysis exhibited increased heterogeneity (*I*^2^ = 61%). In a sensitivity analysis, excluding the study by Edmonston *et al.* [15] reduced the heterogeneity (*I*^2^ = 0%) while maintaining a significant effect [OR 3.08 (95% CI 1.96–4.85), *P* < .00001].

**Figure 5:**
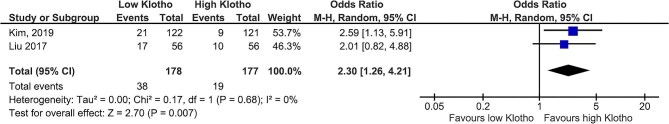
Klotho values and the risk of ESKD or KRT.

**Figure 6:**
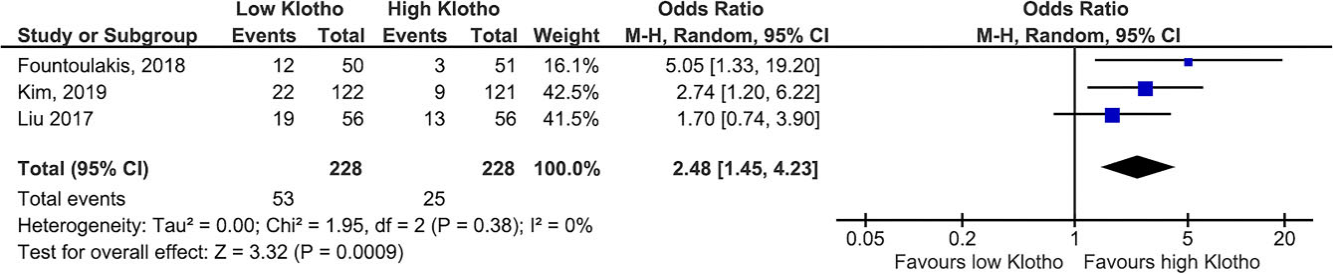
Klotho values and CKD progression.

Data on the impact of klotho on the risk of ESKD or KRT were extracted and analysed from two studies. Despite the limitation of a small sample size, low klotho values were associated with a significantly increased risk of ESKD or KRT [OR 2.30 (95% CI 1.26–4.21), *P* = .007] (Fig. 5). Additionally, the risk of CKD progression was significantly higher in patients with lower klotho levels [OR 2.48 (95% CI 1.45–4.23), *P* = .0009] (Fig. 6).

**Figure 7:**
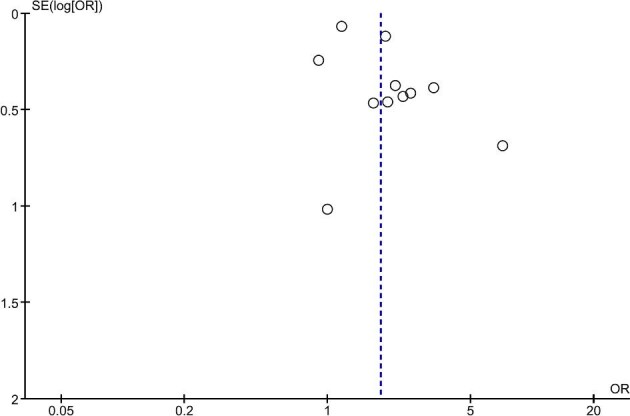
Funnel plot: all-cause mortality.

**Figure 8:**
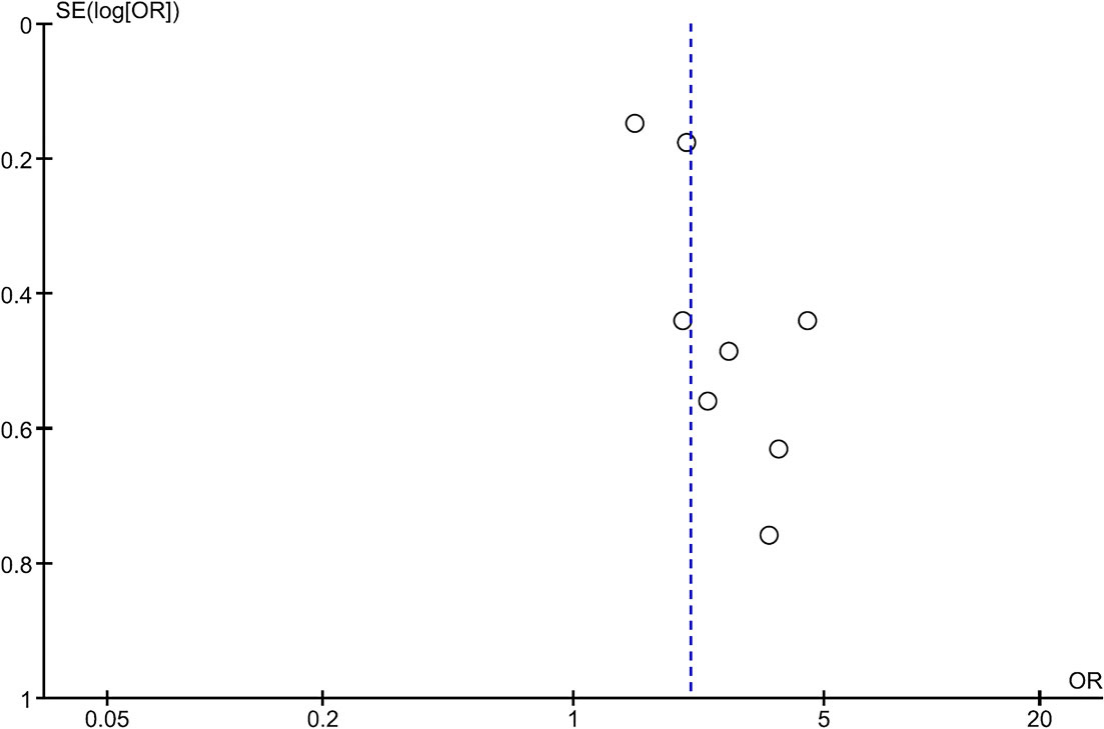
Funnel plot: cardiovascular mortality.

The Newcastle–Ottawa Scale was used to appraise the quality of analysed studies, as presented in Supplementary Table S2. Publication bias was assessed and displayed using funnel plots for studies investigating all-cause mortality and cardiovascular mortality (Figs. 7 and 8, respectively).

The Egger test for funnel plot asymmetry was performed to evaluate the presence of publication bias in the present meta-analysis (for studies evaluating all-cause mortality). The test yielded a *P*-value of .0557, which is slightly above the conventional significance threshold. Therefore, there is no statistically significant evidence to indicate the presence of publication bias in this analysis.

## DISCUSSION

This meta-analysis study provides a large-scale and comprehensive analysis of current literature regarding the association between serum klotho levels and adverse cardiovascular or renal outcomes. We have demonstrated that lower serum klotho levels are linked to a higher risk for all-cause mortality, cardiovascular mortality and adverse renal outcomes including CKD progression or progression into ESKD or KRT requirement among CKD patients in a statistically significant manner.

Similarly, another systematic review and meta-analysis involving a total of six prospective cohort studies conducted in 2020 by Charoenngam *et al.* [6] reported a significant association between lower serum klotho levels and all-cause mortality among CKD patients [RR 1.88 (95% CI 1.29–2.74)]. Moreover, a 2019 meta-analysis involving eight cohort studies with a total of 3586 patients illustrated a considerable association between lower serum klotho levels and adverse kidney outcomes [RR 1.65 (95% CI 1.19–2.26)] [6], aligning with another 2020 meta-analysis conducted by Wang *et al.* [16]. Even though such association has been reported in previous large-scale meta-analyses, our study contributes greatly to the current literature by providing the latest and most comprehensive analysis regarding the prognostic role of serum klotho levels among CKD patients. The exact underlying mechanisms of such protective effects of klotho on cardiovascular and renal health are unclear, but multiple hypotheses have been postulated. First, serum α-klotho has anti-fibrotic, anti-thrombotic and anti-inflammatory properties on vascular endothelium that may regulate endothelial dysfunction, as evident from multiple *in vivo* and *in vitro* studies [17, 18]. Such anti-inflammatory and anti-fibrotic properties are mediated via the inhibition of insulin/insulin-like growth factor-1 along with the downstream pathway of phosphatidylinositol 3-kinase/serine-threonine kinase and transforming growth factor-β signalling pathways [19]. Moreover, elevated levels of α-klotho have been associated with suppression of pro-inflammatory cytokine release, including interleukin-6 and tumour necrosis factor-α [19]. Second, klotho is a major regulator of calcium and phosphorus homeostasis by preventing cardiovascular calcification associated with CKD mineral and bone disorder [20]. The inverse correlation between serum klotho levels and coronary calcium score among CKD patients has been established in a few cohort studies [21, 22]. Third, α-klotho exerts various renoprotective actions, as systemic administration of exogenous klotho has led to attenuation of glomerular and tubular injury in animal models of ischaemic or immune-complex renal injury [23–25]. Such beneficial effects have also been demonstrated in animal models of CKD in which systemic bolus supplementation of soluble klotho protein has led to attenuation of CKD progression without any major adverse effects, although it is unclear whether such a therapeutic approach is applicable to clinical practice [26, 27]. Lastly, klotho deficiency has been associated with cellular senescence and renal fibrosis that is mostly attributed to epithelial-to-mesenchymal transition [28]. Nonetheless, potential confounding factors that lead to both klotho deficiency and poor renal or cardiovascular outcomes, such as hypertension, atherosclerosis, smoking or physical inactivity, may complicate the evaluation of such association [29, 30].

As a decrease in serum klotho levels has been linked to poor clinical outcomes in terms of renal and cardiovascular events, strategies aimed at inducing an increase in serum klotho levels appear to be a valid therapeutic option. However, such therapeutic approaches are primarily based on animal models, with limited data on human subjects. Administration of exogeneous klotho protein or inducing endogenous production has led to promising outcomes in animal models, including reduction in the progression of acute kidney injury to CKD or attenuation of ischaemic renal injury [31, 32]. Multiple pharmacotherapeutic approaches have also been linked to an increase in endogenous production of klotho protein, including renin–angiotensin–aldosterone system blockers, sodium–glucose co-transporter-2 inhibitors, mammalian target of rapamycin inhibitors and statins [33]. Moreover, studies investigating gene therapy targeting klotho protein in the management of neurodegenerative disorders and diabetes mellitus have been conducted [34–36], although there is a clear need for multiple large-scale therapeutic clinical trials for a better understanding of the role of klotho in the management of CKD.

Even though our meta-analysis provides large-scale comprehensive evidence regarding the protective effects of klotho on cardiovascular and renal parameters, this study is not without considerable limitations. First, we have not performed an analysis regarding the potential confounding factors that may affect serum klotho levels and cardiovascular or renal outcomes, including hypertension, atherosclerotic cardiovascular disorders, physical inactivity and smoking. Moreover, the inconsistency across studies in reporting potential confounding variables for both low and high klotho levels constitutes a significant barrier to performing uniform adjustments without excluding a substantial part of the available data. Such exclusions could introduce bias, limit the representativeness of the analysis and reduce the generalizability of the findings. Second, there is considerable heterogeneity between studies included in this meta-analysis, including baseline characteristics of the involved patients that may complicate the evaluation of clinical outcomes. Lastly, there may be potential publication bias, reducing the number of publications with no statistically significant association between serum klotho levels and certain clinical outcomes. Nevertheless, as this meta-analysis provides clear evidence of the protective effects of klotho on cardiovascular and renal outcomes, there is a clear need for clinical studies investigating its potential therapeutic applications.

## Supplementary Material

sfae255_Supplemental_Files

## Data Availability

No new data were generated or analysed in support of this research.
