# Supplementary material for: The association between klotho and kidney and cardiovascular outcomes: a comprehensive systematic review and meta-analysis
Source: Clin Kidney J. 2024 Aug 23;17(9):sfae255. doi: 10.1093/ckj/sfae255 (PMC11398896; doi:10.1093/ckj/sfae255)
Supplement: sfae255_Supplemental_Files [file sfae255_supplemental_files.zip › Supplementary Figure S1. PRISMA - The association between Klotho and prognostic o....docx]

Studies from databases/registers **(n = 2827)**

References from other sources **(n =6)**

Citation searching (n =6)

**Identification**

Studies included in review **(n = 14)**

Studies excluded **(n = 989)**

Studies not retrieved **(n = 0)**

Studies assessed for eligibility **(n = 186)**

Studies sought for retrieval **(n = 186)**

Studies screened **(n = 1175)**

Studies excluded **(n = 172)**

Wrong outcomes (n = 42)

Wrong study design (n = 56)

No full-text available (n = 14)

Wrong patient population (n = 11)

Insufficient reporting of data (n = 9)

Reporting other measures of Klotho (n = 7)

Do not provide an original research (e.g. commentaries, reviews) (n = 33)

References removed **(n = 1658)**

Duplicates identified manually (n = 52)

Duplicates identified by Covidence (n = 1606)

**Screening**

**Included**
