# Supplementary material for: The association between klotho and kidney and cardiovascular outcomes: a comprehensive systematic review and meta-analysis
Source: Clin Kidney J. 2024 Aug 23;17(9):sfae255. doi: 10.1093/ckj/sfae255 (PMC11398896; doi:10.1093/ckj/sfae255)
Supplement: sfae255_Supplemental_Files [file sfae255_supplemental_files.zip › Supplementary Table S1. Search strategy and keywords_klotho (1).docx]

**Supplementary Table S1**. Search strategies and keywords employed for systematic literature search, along with the corresponding number of records retrieved from each database.

| **Total Records: 2827** |
| --- |
| **After Delete of Duplicate Records: 1231 (by Endnote)*** |
| **PubMed (n=3,810)** |
| 4 (("Klotho Proteins"[Mesh] OR klotho) AND ("Renal Insufficiency, Chronic"[Mesh] OR "Chronic Renal Insufficiencies" OR "Chronic Kidney Insufficiency" OR "chronic kidney disease" OR "chronic renal disease" OR "chronic kidney failure" OR "Renal Dialysis"[Mesh] OR "Renal Dialy*" OR "diabetic kidney" OR "end-stage renal disease")) AND ("CKD progression" OR "chronic kidney disease progression" OR mortality OR death OR survival OR "renal replacement therapy" OR "dialysis initiation" OR "GFR decline" OR "glomerular filtration rate" OR "kidney function" OR "cardiovascular" OR mace OR "metabolic syndrome" OR "cardiometabolic" OR hemodialy*) ("Klotho Proteins"[MeSH Terms] OR ("klotho"[All Fields] OR "klotho s"[All Fields] OR "klothos"[All Fields])) AND ("renal insufficiency, chronic"[MeSH Terms] OR "Chronic Renal Insufficiencies"[All Fields] OR "Chronic Kidney Insufficiency"[All Fields] OR "chronic kidney disease"[All Fields] OR "chronic renal disease"[All Fields] OR "chronic kidney failure"[All Fields] OR "Renal Dialysis"[MeSH Terms] OR "renal dialy*"[All Fields] OR "diabetic kidney"[All Fields] OR "end-stage renal disease"[All Fields]) AND ("CKD progression"[All Fields] OR "chronic kidney disease progression"[All Fields] OR ("mortality"[MeSH Terms] OR "mortality"[All Fields] OR "mortalities"[All Fields] OR "mortality"[MeSH Subheading]) OR ("death"[MeSH Terms] OR "death"[All Fields] OR "deaths"[All Fields]) OR ("mortality"[MeSH Subheading] OR "mortality"[All Fields] OR "survival"[All Fields] OR "survival"[MeSH Terms] OR "survivability"[All Fields] OR "survivable"[All Fields] OR "survivals"[All Fields] OR "survive"[All Fields] OR "survived"[All Fields] OR "survives"[All Fields] OR "surviving"[All Fields]) OR "renal replacement therapy"[All Fields] OR "dialysis initiation"[All Fields] OR "GFR decline"[All Fields] OR "glomerular filtration rate"[All Fields] OR "kidney function"[All Fields] OR "cardiovascular"[All Fields] OR ("myristica"[MeSH Terms] OR "myristica"[All Fields] OR "mace"[All Fields]) OR "metabolic syndrome"[All Fields] OR "cardiometabolic"[All Fields] OR "hemodialy*"[All Fields]) 653  3 "CKD progression" OR "chronic kidney disease progression" OR mortality OR death OR survival OR "renal replacement therapy" OR "dialysis initiation" OR "GFR decline" OR "glomerular filtration rate" OR "kidney function" OR "cardiovascular" OR mace OR "metabolic syndrome" OR "cardiometabolic" OR hemodialy* "CKD progression"[All Fields] OR "chronic kidney disease progression"[All Fields] OR "mortality"[MeSH Terms] OR "mortality"[All Fields] OR "mortalities"[All Fields] OR "mortality"[MeSH Subheading] OR "death"[MeSH Terms] OR "death"[All Fields] OR "deaths"[All Fields] OR "mortality"[MeSH Subheading] OR "mortality"[All Fields] OR "survival"[All Fields] OR "survival"[MeSH Terms] OR "survivability"[All Fields] OR "survivable"[All Fields] OR "survivals"[All Fields] OR "survive"[All Fields] OR "survived"[All Fields] OR "survives"[All Fields] OR "surviving"[All Fields] OR "renal replacement therapy"[All Fields] OR "dialysis initiation"[All Fields] OR "GFR decline"[All Fields] OR "glomerular filtration rate"[All Fields] OR "kidney function"[All Fields] OR "cardiovascular"[All Fields] OR "myristica"[MeSH Terms] OR "myristica"[All Fields] OR "mace"[All Fields] OR "metabolic syndrome"[All Fields] OR "cardiometabolic"[All Fields] OR "hemodialy*"[All Fields] 4,509,708  2 "Renal Insufficiency, Chronic"[Mesh] OR "Chronic Renal Insufficiencies" OR "Chronic Kidney Insufficiency" OR "chronic kidney disease" OR "chronic renal disease" OR "chronic kidney failure" OR "Renal Dialysis"[Mesh] OR "Renal Dialy*" OR "diabetic kidney" OR "end-stage renal disease" "renal insufficiency, chronic"[MeSH Terms] OR "Chronic Renal Insufficiencies"[All Fields] OR "Chronic Kidney Insufficiency"[All Fields] OR "chronic kidney disease"[All Fields] OR "chronic renal disease"[All Fields] OR "chronic kidney failure"[All Fields] OR "Renal Dialysis"[MeSH Terms] OR "renal dialy*"[All Fields] OR "diabetic kidney"[All Fields] OR "end-stage renal disease"[All Fields] 272,397  1 "Klotho Proteins"[Mesh] OR klotho "Klotho Proteins"[MeSH Terms] OR "klotho"[All Fields] OR "klotho s"[All Fields] OR "klothos"[All Fields] 3,810 |
| **Cochrane Library (n=66)** |
| #1 klotho 219  #2 "Chronic Renal Insufficiencies" OR "Chronic Kidney Insufficiency" OR "chronic kidney disease" OR "chronic renal disease" OR "chronic kidney failure" OR Renal NEXT Dialy* OR "diabetic kidney" OR "end-stage renal disease" 22007  #3 "CKD progression" OR "chronic kidney disease progression" OR mortality OR death OR survival OR "renal replacement therapy" OR "dialysis initiation" OR hemodialy* OR "GFR decline" OR "glomerular filtration rate" OR "kidney function" OR "cardiovascular" OR MACE OR "metabolic syndrome" OR "cardiometabolic" 376107  #4 #1 AND #2 AND #3 66 |
| **Scopus (n=510)** |
| TITLE-ABS ( "klotho" ) AND TITLE-ABS ( "Chronic Renal Insufficiencies" OR "Chronic Kidney Insufficiency" OR "chronic kidney disease" OR "chronic renal disease" OR "chronic kidney failure" OR "Renal Dialy*" OR "diabetic kidney" OR "end-stage renal disease" ) AND TITLE-ABS ( "CKD progression" OR "chronic kidney disease progression" OR mortality OR death OR survival OR "renal replacement therapy" OR "dialysis initiation" OR "GFR decline" OR "glomerular filtration rate" OR "kidney function" OR "cardiovascular" OR "MACE" OR "metabolic syndrome" OR "cardiometabolic" OR hemodialy* )  510 |
| **Web of Science (n=819)** |
| 1: TS=( "klotho" ) Date Run: Tue Jun 04 2024 15:42:25 GMT+0300 (GMT+03:00) Results: 5216  2: TS=("Chronic Renal Insufficiencies" OR "Chronic Kidney Insufficiency" OR "chronic kidney disease" OR "chronic renal disease" OR "chronic kidney failure" OR "Renal Dialy*" OR "diabetic kidney" OR "end-stage renal disease" ) Date Run: Tue Jun 04 2024 15:44:37 GMT+0300 (GMT+03:00) Results: 151518  3: TS=("CKD progression" OR "chronic kidney disease progression" OR mortality OR death OR survival OR "renal replacement therapy" OR "dialysis initiation" OR "GFR decline" OR "glomerular filtration rate" OR "kidney function" OR "cardiovascular" OR "MACE" OR "metabolic syndrome" OR "cardiometabolic" OR hemodialy*) Date Run: Tue Jun 04 2024 16:02:36 GMT+0300 (GMT+03:00) Results: 4476040  4: #3 AND #2 AND #1 Date Run: Tue Jun 04 2024 16:02:39 GMT+0300 (GMT+03:00) Results: 819 |
| **CINAHL (n=63)** |
| S1 "klotho" AND ( "Chronic Renal Insufficiencies" OR "Chronic Kidney Insufficiency" OR "chronic kidney disease" OR "chronic renal disease" OR "chronic kidney failure" OR "Renal Dialy*" OR "diabetic kidney" OR "end-stage renal disease" ) AND ( "CKD progression" OR "chronic kidney disease progression" OR mortality OR death OR survival OR "renal replacement therapy" OR "dialysis initiation" OR "GFR decline" OR "glomerular filtration rate" OR "kidney function" OR "cardiovascular" OR "MACE" OR "metabolic syndrome" OR "cardiometabolic" OR hemodialy* ) Expanders - Also search within the full text of the articles; Apply equivalent subjects  Search modes - Boolean/Phrase Interface - EBSCOhost Research Databases  Search Screen - Advanced Search  Database - CINAHL Plus with Full Text 163 |
| **Ovid MEDLINE(R) and Epub Ahead of Print, In-Process, In-Data-Review & Other Non-Indexed Citations, Daily and Versions <1946 to June 03, 2024> (n=616)** |
| 1 Klotho Proteins/ or klotho.mp. 3809  2 "Chronic Renal Insufficiencies".mp. or Renal Insufficiency, Chronic/ 39220  3 "Chronic Kidney Insufficiency".mp. 251  4 "chronic kidney disease".mp. 82462  5 "chronic renal disease".mp. 3890  6 "chronic kidney failure".mp. or Kidney Failure, Chronic/ 103281  7 Renal Dialysis/ or "Renal Dialy*".mp. 105739  8 "diabetic kidney".mp. 6912  9 "end-stage renal disease".mp. 40556  10 2 or 3 or 4 or 5 or 6 or 7 or 8 or 9 256089  11 "CKD progression".mp. 2652  12 "chronic kidney disease progression".mp. 629  13 mortality.mp. or Mortality/ 1465211  14 death.mp. or Death/ 1026760  15 Survival/ or survival.mp. 1568387  16 "renal replacement therapy".mp. or Renal Replacement Therapy/ 20623  17 "dialysis initiation".mp. 1267  18 "GFR decline".mp. 538  19 "glomerular filtration rate".mp. or Glomerular Filtration Rate/ 79712  20 "kidney function".mp. 53634  21 "cardiovascular".mp. or Cardiovascular Diseases/ or Cardiovascular System/ 723805  22 "MACE".mp. 12953  23 "metabolic syndrome".mp. or Metabolic Syndrome/ 73855  24 "cardiometabolic".mp. 22073  25 hemodialy*.mp. 80238  26 11 or 12 or 13 or 14 or 15 or 16 or 17 or 18 or 19 or 20 or 21 or 22 or 23 or 24 or 25 3948295  27 1 and 10 and 26 616 |

*EndNote automatically removes the majority of the duplicate records and the remaining duplicates were manually removed by the authors. The count of duplicate records in this row reflects those automatically identified through EndNote.
