# Supplementary material for: The association between klotho and kidney and cardiovascular outcomes: a comprehensive systematic review and meta-analysis
Source: Clin Kidney J. 2024 Aug 23;17(9):sfae255. doi: 10.1093/ckj/sfae255 (PMC11398896; doi:10.1093/ckj/sfae255)
Supplement: sfae255_Supplemental_Files [file sfae255_supplemental_files.zip › Supplementary Table S2.docx]

**Supplementary Table S2:** Quality assessment of included studies using the Newcastle-Ottawa Scale for cohort studies

| **Study** | **Study**  **Design** | **Selection** | | | | **Comparability** | **Outcome** | | | **Total  Score** |
| --- | --- | --- | --- | --- | --- | --- | --- | --- | --- | --- |
|  |  | *Representative of exposed cohort* | *Selection of non-exposed cohort* | *Ascertainment of exposure* | *Outcome not present at start* | *On the basis of design or analysis* | *Assessment of outcome* | *Follow-up >1 year* | *Follow-up adequate* |  |
| Buiten, 2014 | Cohort | * | * | * | * | * | * |  |  | **6** |
| Cai, 2021 | Retrospective cohort | * | * | * | * | * | * | * | * | **8** |
| Chen, 2024 | Retrospective Cohort | * | * | * | * | * | * | * | * | **8** |
| Edmonston, 2024 | Prospective cohort | * |  | * | * | * | * | * | * | **7** |
| Fountoulakis, 2018 | Prospective Cohort | * | * | * | * | * | * | * | * | **8** |
| Kim, 2019 | Retrospective Cohort | * | * | * | * | * | * |  |  | **6** |
| Liu, 2017 | Prospective cohort | * |  | * | * | * | * | * | * | **7** |
| Liu, 2022 | Prospective cohort | * | * | * | * | * | * | * | * | **8** |
| Liu, 2024 | Retrospective cohort | * | * | * | * | ** | * | * | * | **9** |
| Martins, 2023 | Prospective cohort | * |  | * | * | * | * | * | * | **7** |
| Memmos, 2019 | Prospective Cohort | * | * | * | * | * | * | * | * | **8** |
| Otani-Takei, 2015 | Prospective Cohort | * | * | * | * | * | * | * | * | **8** |
| Yu, 2020 | Prospective Cohort | * | * | * | * | * | * | * | * | **8** |
| Zheng, 2018 | Prospective Cohort | * | * | * | * | * | * |  | * | **7** |
